# Trunk and hip muscle recruitment patterns during the prone leg extension following a lateral ankle sprain: A prospective case study pre and post injury

**DOI:** 10.1186/1746-1340-14-4

**Published:** 2006-02-27

**Authors:** Gregory J Lehman

**Affiliations:** 1 Department of Graduate Studies, Canadian Memorial Chiropractic College, Toronto, ON, Canada

## Abstract

**Background and case presentation:**

The prone leg extension (PLE) is commonly used to identify dysfunction of muscle recruitment patterns. The prone leg extension is theorized to identify proximal muscle disturbances which are a result of distal injury or dysfunction (i.e. an ankle sprain). This case study compares the trunk and hip muscle (bilateral lower erector spine, ipsilateral hamstring and ipsilateral gluteus maximus) timing during a PLE of a 27 year old female runner during a healthy state (pre ankle sprain) and 2 and 8 weeks post ankle sprain.

**Results and discussion:**

The gluteus maximus muscle onsets at 8 weeks post injury appeared to occur earlier compared with 2 weeks post injury. The Right Erector Spinae at 8 weeks post injury was also active earlier compared with the participant's non-injured state. A large degree of variability can be noted within trials on the same day for all muscle groups.

**Conclusion:**

An acute ankle injury did not result in a delay in gluteus maximus muscle activation. The utility of the prone leg extension as a clinical and functional test is questionable due to the normal variability seen during the test and our current inability to determine what is normal and what is dysfunctional.

## Background

The prone leg extension (PLE) test is used by therapists who manually palpate the posterior muscles and observe trunk kinematics in an attempt to identify deviations from a supposed optimal. A dysfunctional firing pattern is assumed to exist when postural muscles (hamstrings and erector spinae) initiate the movement and a delay in gluteus maximus firing is evident [1]. As well, inconsistent or altered firing patterns are assumed to be indicative of dysfunction. While dysfunctions in the trunk are hypothesized to alter the optimal firing pattern, distal dysfunction (ankle sprain) is also theorized to influence the proximal order of optimal firing patterns [2]. An ankle sprain or lower leg injury is theorized to result in "imbalances" in the firing pattern of proximal muscles due to the dysfunction of distal segments. This distal dysfunction is assumed to affect the proximal segments via the kinetic chain from distal to proximal during weight bearing.

Bullock-Saxton et al [2] investigated the influence of a previous ankle sprain on the firing order and timing of the posterior trunk and leg muscles during the PLE. The authors found a significant difference in the onset of the gluteus maximus activity (delayed onset) in the group with previous ankle sprains compared with the control group. Of note was the lack of support for the theorized ideal pattern of muscle onset in the control group.

The Bullock-Saxton study [2] was a cross sectional study that is inherently unable to determine if an ankle sprain is a **cause **of disturbances in the muscle firing pattern during the PLE. Presented in this case study is a longitudinal quantitative assessment of the muscle onset timing of posterior trunk and leg muscles during the prone leg extension following an acute lateral ankle sprain. This case study will specifically address whether an acute ankle sprain precedes a delay in the muscle activation of the ipsilateral Gluteus Maximus muscle.

## Case presentation

### Overview

This case study is a product of serendipity. A 27 year old female runner was initially part of an experimental study documenting the muscle onset timing of the posterior leg and trunk muscles during the prone leg extension. At the time of the study the participant had no leg or spine injuries. The participant was part of the asymptomatic control group. Five months after the completion of the study this participant suffered an inversion right ankle sprain during a rainstorm while leaping into a camping tent filled with children. At 2 and 8 weeks post ankle sprain the PLE extension test was performed while recording the posterior muscle activation EMG to determine if an ankle injury influences the timing of muscle activation during the PLE as compared with an injury-free PLE onset timing pattern.

### Participant characteristics

Twenty seven year old female distance runner (height – 167 cm, weight -58 kg) suffered a right ankle inversion sprain. X rays revealed no fractures. Informed consent to participate in this study was received from the subject. In the initial study the participant read and signed an information and consent form that was approved by the Research Ethics Board of CMCC. For the second half of the study the subject was notified of the risks and benefits of the study which complied with the World Medical Association declaration of Helsinki on the ethical conduct of research using human participants within a private practice and agreed to have her results published.

### Treatment & subjective functional rating

No passive therapy was received by the participant. The participant was encouraged to perform daily weight bearing exercise and range of motion exercises. The participant was running within two weeks of the injury with pain and not at her pre injury level. At two weeks post injury the participant reported that she felt she was functioning at 60% of her optimum. At 8 weeks post injury the participant reported that she was running 95% pain free with little loss of function.

### Experimental protocol

An identical testing protocol was used across the 3 experimental days (pre injury, 2 weeks post injury and 8 weeks post injury). The muscle activity of the right gluteus maximus, bilateral lower erector spinae, and right hamstring muscle groups was recorded during right prone leg extension while lying prone on a manual therapy table. The position of the leg was controlled in all planes (no hip adduction/abduction or internal/external rotation) visually by the experimenter. A rig restricting movement or a kinematic analysis system to ensure an identical movement across trials was not used as this is not similar to what occurs during practice. The control of proper form was limited to a visual assessment as this most resembles clinical practice.

### Data collection hardware characteristics

Disposable bipolar Ag-AgCl disc surface electrodes with a diameter of one cm were adhered bilaterally over the five muscle groups with a centre to centre spacing of 1 cm. Raw EMG was amplified 5000 times. The amplifier has a CMRR of 10,000:1 (Bortec EMG, Calgary AB, Canada). Raw EMG was band pass filtered (10 and 1000 Hz) and A/D converted at 2000 Hz using a National Instruments data acquisition system.

### PLE tasks

The prone leg extension exercise was performed five times during each experimental session. The task required the subject to lie prone at complete rest with no movement while the EMG from each muscle was collected for 5 seconds. The subject then extended their straight right leg approximately six inches off the table. The leg was held isometrically for 3 seconds then lowered to the table.

### EMG processing

The data from each PLE trial was processed in the same manner. The aim of the processing for this study was to determine the order and timing of muscle activation. To determine muscle timing, it is necessary to determine when a muscle is considered active or "on". A muscle was considered "on" when the level of muscle activity was greater than 10% of the peak muscle activity during the prone leg extension. This method of determining muscle onset was used in the previous study by Bullock-Saxton et al [2]. The order of activation can then be determined by classifying each muscle as "on" when its level of activity exceeds that of its predetermined threshold. Muscle activation time (milliseconds-ms) was referenced to the time of activation of the hamstring muscle. For example, positive values (ms) occurred when a muscle's onset occurred before activation of the hamstring muscle group, and negative values indicated that muscle activation occurred after the onset of the hamstring muscle. The onset of muscle activity was determined for each muscle during each repetition of the prone leg lift. Please note that the EMG activity was not normalized to a maximum voluntary contraction (MVC). While MVCs are important in the collection of EMG when there is a need for determining muscle amplitude, this was not necessary in the current study because muscle timing rather than amplitude was measured. Normalization would not influence the muscle onset results.

The data was processed in the following manner: The raw EMG signal was first full wave rectified, then smoothed using a moving average technique which averaged every 100 points of data with an overlap 98 points. The bias was removed from the signal to allow resting activity to be at 0. The peak muscle activity was found and each data point was divided by this peak muscle activity. In this way, muscle onset could be determined by determining the time when the myoelectric signal exceeded 10% of the maximum. The signal was visually inspected to ensure that no artefact occurred or that the results were biologically feasible.

## Results

Table 1 presents the average activation for each muscle before injury, 2 weeks post injury and 8 weeks post injury. There appears to be differences in the activation timing of the gluteus maximus between 2 weeks and 8 weeks post injury – the gluteus maximus onset time was earlier 8 weeks post injury. There was also a significant difference in the activation times of the Right Erector Spinae between the pre injury status and 8 weeks post injury – the RES onset time was earlier at 8 weeks post injury. A large degree of variability can be noted within trials on the same day for all muscle groups.

**Table 1:** Mean (SD) EMG onset and spread (ms)

	**RES**	**LES**	**RGM**	**Spread**
**Healthy Ankle**	137.4 (151.8)	1.6 (56.01)	306.2 (33.18)	333 (26.32)
**2 weeks Post Ankle Sprain**	72 (108.1)	-56.2 (84.3)	462.2 (105.9)	532.6 (121.8)
**8 w Post Ankle Sprain**	-135 (83.4)	-154 (119.2)	153.2 (79.9)	331.4 (135.4)

Values are referenced to the onset of hamstring activation. (-) values indicate that muscle activity occurred before the onset of hamstring activation. The spread is the time difference in ms between the onset of the first muscle activated and that of the last muscle activated.

## Discussion & implications

To the knowledge of the author this is the only prospective case documenting the longitudinal influence of an inversion ankle sprain on muscle activation patterns during the prone leg extension test. The results from this one participant failed to support the idea that lower limb injury leads to a delay in gluteus maximus firing as suggested by the cross sectional study of Bullock-Saxton et al [2].

Conversely, at 8 weeks post ankle sprain there was a decrease in the latency of the gluteus maximus muscle (the muscle fired earlier). This earlier firing is similar to the differences in onset timing of the gluteus medius during lateral ankle perturbations between groups with chronically sprained ankles and controls [6]. Beckman and Buchanan [6] found that the reflexive latency response of the gluteus medius in the group with hypermobile ankles was significantly reduced compared with controls.

The lack of support from this case study and previous research questions the utility of the PLE in determining dysfunctional muscle firing patterns. There is little support in the research literature for the use of the PLE test in assessing muscle onset dysfunction. Further evidence against the use of the PLE in adequately assessing muscle function is found in the conclusions of Pierce and Lee [3] and Lehman et al [4] who both failed to show a consistent pattern of muscle onsets and found high within subject and across subject variability. Lehman et al [4] found that the gluteus maximus was the last muscle active in 13/14 asymptomatic control subjects. This finding is considered to be an indicator of dysfunction but appears to be normal. This delay in Gluteus Maximus firing was also found be Vogt et al [5] in a group of asymptomatic subjects. However, Vogt et al [5] did conclude that a consistent pattern of muscle activation occurred – in disagreement with the findings of Pierce and Lee [3] and Lehman et al [4] and in support of the traditional theory behind the use of the prone leg extension. The weaknesses of the PLE for identifying dysfunction in muscle onset may be further amplified when function is assessed with palpation rather than through electromyography considering the inherent inaccuracies and lack of sensitivity compared with EMG techniques.

An interesting trend in changes in muscle activation was earlier muscle activation of the erector spinae activity. Before the injury both erector spinae were active after the hamstring group. Eight weeks post injury the erector spinae preceeded hamstring activation by more than 130 ms. This earlier activation was also seen in the contralateral Erector Spinae at 2 weeks post injury (56.2 ms before hamstring activation). Bullock-Saxton et al [2] discussed the idea that earlier activation of the erector spinae (postural muscles) may be a compensation for a delay or weakness in the Gluteus Maximus. In this case study the earlier activation **was not **associated with a delay in the gluteus maximus activity. The Gluteus Maximus at 8 weeks post injury fired earlier than 2 weeks post injury and showed a trend to firing earlier than in a healthy state.

Another weakness of the PLE test is the high variability between trials within one individual (as seen in the current case study) and across individuals. This variability appears normal and limits the use of EMG in producing data which can identify individuals with dysfunction [3,4]. Last, the differences in muscle EMG onsets across the testing days appear significant but may not be palpable to the human hand. The activation spread across muscles was between 300–500 ms. It is questionable whether the hand or the eye can discern a difference of 200 ms especially considering the degree of adipose tissue above the gluteus maximus that may act as a filter to further inhibit an accurate subjective assessment of muscle activation timing.

While this case study failed to support the proposal that distal dysfunction may result in proximal dysfunction manifesting as aberrant muscle onset timing of the gluteus maximus during the PLE, the PLE as a clinical assessment test may still have value. The only means of assessing the PLE has been via muscle onset timing with electromyography. As stated earlier the trunk and hip kinematics of the participant are observed during the performance of the movement. No research to date has measured the kinematics with the aim of identifying and quantifying optimal and dysfunctional movement patterns (as opposed to the motor patterns assessed with electromyography). Quantifying these kinematics may lead to determining the discriminant ability of this test for the identification of dysfunction. The PLE may have more clinical utility as a crude test of function – minor differences in muscle onsets may have no clinical value and may not be easily detected by observation; however, major dysfunctions in muscle activation (i.e. no observable gluteus maximus contraction) would be more easily detected and may be of more clinical value. For example, during the test a patient's buttock may remain flaccid. This is easily seen and may be indicative of inhibition and possible dysfunction. The use of the PLE as a gross detector of dysfunction may be more supportable than being used to questionably detect subtle differences in firing patterns that are within 200 ms of each other.

## Conclusion

An acute ankle injury did not result in a delay in gluteus maximus muscle activation. The utility of the prone leg extension as a clinical and functional test is questionable due to the normal variability seen during the test and our current inability to determine what is normal and what is dysfunctional.

## Competing interests

The author(s) declare that they have no competing interests.
